# Challenges and Resilience-Building: A Narrative Inquiry Study on a Mid-Career Chinese EFL Teacher

**DOI:** 10.3389/fpsyg.2021.758925

**Published:** 2021-10-12

**Authors:** Lina Xue

**Affiliations:** School of College English Teaching and Research/Center for Second Language Writing Research, Henan University, Kaifeng, China

**Keywords:** resilience-building, EFL teacher, mid-career teacher, narrative inquiry, challenges

## Abstract

With a high rate of attrition and burnout of teachers as a global concern, teacher resilience has become a trendy topic in the research of their professional development as one of the pillars of positive psychology (positive character traits). However, the literature reveals that little research has been done on the mid-career teachers in the Chinese context, especially on how resilience may be nurtured, sustained, or eroded over time. Focusing on a mid-career EFL female teacher (the author) in China as a case study, this longitudinal self-reflective study employs a narrative inquiry to investigate the challenges that the experienced teacher was encountered with and to depict her trajectories of resilience-building by fleshing out the interaction between challenges, resources, and coping strategies in her three different scenarios. “Hard data,” such as teaching journals, reflective field notes, and messages with students were collected and analyzed inductively by using thematic analysis, and “soft data,” like memory was also referred to. The findings unfolded challenges confronting the experienced teacher peculiar to the Chinese context and charted a detailed bumpy journey of resilience building in three phases, accompanied by her growing emotional, intellectual, and psychological capacities. Implications are drawn out for teacher resilience building, school leaders, and policymakers.

## Introduction

A body of research has proved that teaching has become one of the most demanding and stressful professions in any field in the 21st century (Travers and Cooper, 1996; Nash, 2005; Gu and Day, 2007; Derakhshan et al., 2020a). High workload, disruptive students’ behavior, role conflict, ambiguity, and other risk factors have made teachers’ job burnout and attrition a global concern (Gu and Day, 2007; Gu and Li, 2013; Day, 2017; Fathi et al., 2021). Instead of investigating negative factors for “who have left” as the traditional psychological science did, the research of resilience as a topic of positive psychology is initiated to unearth protective factors for “who have stayed” (Beltman et al., 2011). Resilience research in SLA is a recent area of investigation in the positive psychology shift (Seligman and Csikszentmihalyi, 2000; MacIntyre and Mercer, 2014; MacIntyre et al., 2016; Budzińska and Majchrzak, 2021; Wang et al., 2021). It has been illuminated by the resilience theories in the field of psychology including Resiliency Model of Richardson et al. (1990), and the broaden-and-build theory of Fredrickson (2004). Although as a nascent area, resilience studies in SLA have documented abundant effective strategies to foster and maintain teachers’ motivation and commitment in the context of Britain (Gu and Day, 2007, 2013; Greenier et al., 2021), Australia (Mansfield et al., 2014; Mansfield, 2021), America (Mansfield, 2021), and Iran (Derakhshan et al., 2020b).

An overview of previous studies reveals that almost all the research on teacher resilience were conducted in Western contexts (Fan et al., 2021; Wang et al., 2021). Few studies focus on the risk factors and resilience measures adopted by foreign language teachers in the Chinese context, which is characterized with examination-oriented education and national reform policy (Fan et al., 2021). A further point is worth noting that fewer studies target mid-career or late-career teachers, who face their challenges different from those novice teachers, and the importance of paying special attention to this population of teachers has been increasingly recognized by researchers (Gu and Day, 2013; Day, 2017; Fan et al., 2021; Jin et al., 2021). It is reasonable to pay attention to novice teachers since they are in a typical period of disruption, struggling to adapt to new roles and constructing identities. Equally, attention should be paid to mid-career and late-career teachers whose capacity for resilience may become eroded to the extent that survival in the classroom rather than pursuing professional development becomes the main concern (Gu and Day, 2013; Jin et al., 2021). As a culturally embedded conception, resilience building is not homogeneous or the context matters (MacIntyre and Mercer, 2014; Gu, 2021), so the panoramic research of the novice teachers as well as mid-career teachers in both western and eastern contexts will deepen our understanding of this construct, and benefit the quality education ultimately. In general, positive psychology has not focused on the individual very well in the previous literature, from which it has suffered for a long time (MacIntyre and Mercer, 2014; MacIntyre, 2021). Therefore, this qualitative case study aims to contribute to the full picture of this domain by exploring the challenges confronting an experienced Chinese EFL teacher and depicting her trajectories of resilience building in the complex teaching circumstances peculiar to China.

It should be noted that this study is exploratory and interpretive, so the generalizability of the findings will be limited (Barkhuizen et al., 2014). By providing a thick description of the case and a close analysis of the narrative, the current study aims to document the complexity of the resilience-building journey and to deepen our understanding of the challenges confronting the experienced teacher in particular (Xie and Derakhshan, 2021). Hopefully, this study will lead to more studies on the resilience and the professional development of experienced teachers.

## Literature Review

Resilience is a relative, multi-faceted, and developmental construct (Gu and Day, 2007, 2013; Mansfield et al., 2012; Gu and Li, 2013). Studies of resilience were originated in the field of psychology to tackle children’s misbehaviors like drug abuse, alcohol abuse, or mental disorders after exposure to serious hazards (Richardson et al., 1990; Rutter, 1999; Fredrickson, 2004). As a nascent area of investigation, teacher resilience has been conceptualized in the literature in a range of ways. Initially, resilience was defined as the capacity of individuals to adapt and thrive in confronting adverse conditions (Masten et al., 1990; Richardson et al., 1990). Researchers then gradually discovered instead of being “innate attribute,” resilience was constructed by the dynamic interaction between individual, contextual, and organizational factors (Beltman et al., 2011; Doney, 2013). In the same vein, Yonezawa et al. (2011) regarded resilience as the conflation of resilient characteristics of teachers and the environmental supports. In order to create a deeper, holistic notion of teacher resilience, Beltman (2020) chronologically outlined the four shifting perspectives, namely the person-focused, the process-focused, the context-focused, and the system-focused perspective. Based on it, Beltman has further broadened this concept and stated that teacher resilience was a capacity, a process, and also an outcome. According to Beltman (2020), resilience involves the capacity of an individual teacher to harness personal and contextual resources to navigate through challenges, the dynamic process whereby characteristics of individual teachers and of their personal and professional contexts interact over time as teachers use particular strategies, to enable the outcome of a teacher who experiences professional engagement and growth, commitment, enthusiasm, satisfaction, and wellbeing. Beltman echoed studies in the field of psychology by regarding resilience as a dynamic process of development that occurs over time (Richardson et al., 1990; Bobek, 2002). According to Richardson et al. (1990), the resiliency model encompasses three critical stages: Firstly, an individual’s organized state is broken by challenges, stressors, or risk factors; then the individual will struggle to reorganize his or her life and regain equilibrium from the traumatic experiences; eventually, he or she will be more adept at protective factors or coping strategies. Obviously, resilience is a growing process of different stages, through dynamic interaction between individual circumstances and social factors, and eventually one will acquire commitment, satisfaction, and well-being. This study has adopted the concept of teacher resilience of Beltman (2015, 2020) and highlighted how resilience is developed and how the individual teacher’s agency counts in coping with various challenges outside and inside. Meanwhile, this study would also use the Resilience Model (Richardson et al., 1990) as the theoretical framework to fully depict the trajectories of how the experienced teacher navigates the changing and challenging circumstances and builds her resilience at the corresponding stage.

An overview of the previous studies on teacher resilience mainly involves three aspects: risk factors, protective factors, and coping strategies. Risk factors, stressors, or challenges are a prerequisite for initiating resilience building (Fan et al., 2021). Studies report personal and contextual risk factors confronting teachers in the western context. On the individual level, a sense of low self-esteem and isolation, the lack of experience and confidence, role conflict and ambiguity, and inadequate language competence are often documented (Travers and Cooper, 1996; Kyriacou, 2001; Howard and Johnson, 2004; Mousavi, 2007; Day, 2008). On the contextual level, predominant challenges are disruptive students, uncooperative parents, poor working conditions (workload and salary), and lack of collegial and organizational support (Day, 2008; Yonezawa et al., 2011; Gu and Day, 2013; Beltman, 2015).

While resilience presumes the existence of risk factors, the resilience-building relies heavily on resources (or protective factors) and strategies with which one can “bounce back.” As for personal resources, important factors identified are mainly related to motivation and emotion, such as efficacy, a sense of moral purpose, agency, stroke, credibility, and optimism (Gu and Day, 2007; Tait, 2008; Fathi et al., 2020, 2021; Fan et al., 2021; Pishghadam et al., 2021). According to Fredrickson (2004), positive emotions including joy, contentment, interest, and love could broaden people’s momentary thought-action repertoires, and build their enduring personal resources. The experiencing of positive emotions was transformative to people by making them more creative, knowledgable, resilient, socially integregrated, and healthy (Fredrickson, 2004). Contextual resources especially refer to important relationships both within and outside the working context, such as supporting family, trusting colleges, empowering leaders, and resilience-promoting school cultures (Howard and Johnson, 2004; Gu and Day, 2007; Gu, 2014; Jin et al., 2021). Studies reveal strategies to buffer challenges and promote well-being adopted by teachers include problem-solving, work-life balancing, professional learning and reflecting, and emotional boundaries setting (Hong, 2012; Johnson et al., 2014; Tait, 2008). Fan et al. (2021) revealed four strategies that novice college teachers adopted to cope with hardships, namely perceiving risks as opportunities, taking initiatives to motivate students, seeking help from the social network, and keeping professional learning. It is noteworthy that although researchers have long accepted the nature of teacher resilience is interactive, dynamic, and developmental, most studies still rest upon investigating and recording risk factors, protective factors, and strategies in a static way, some even equate resilience studies with the exploration of protective factors (Kumpfer, 2002), and very few go beyond the static description to explore teachers’ detailed, specific struggling and resilience building journey (Fan et al., 2021). Despite the seemingly dynamic perspective of studies on coping strategies, most of them just explore the repertoire of coping actions adopted by teachers without chronologically revealing their resilience growing detours in the midst of interaction between challenges and resources.

The previous studies contribute a lot to our understanding of the multi-dimensional construct, yet teacher resilience, to a large extent, is also fluctuating and relevant to different scenarios and career phases (Rutter, 1999; Gu and Day, 2007, 2013). Until now, most of the studies have been conducted in the western contexts, and scattered studies have been carried out in the Chinese context (Gu and Li, 2013; Fan et al., 2021). Among the few studies relevant to Chinese teachers, Gu and Li (2013) explored how 568 primary and secondary teachers sustained their resilience and commitment in China’s educational system, and Fan et al. (2021) targeted seven novice foreign language teachers to investigate risk factors and coping strategies. To our knowledge, no study to date has researched challenges confronting the experienced or mid-career EFL teachers and how they respond, reflect, and rebuild resilience in the midst of reforms and changes peculiar to China. With a huge population of mid-career teachers and shifting educational policies and reforms in China, it would be fruitful to probe into their resilience-building trajectories and offer insights to help teachers sustain their motivation, commitment, and effectiveness. Therefore, this study has explored the challenges the mid-career Chinese EFL teacher (the author) was confronted with in her different working phases, and how she juggled initially and gradually became adept at integrating personal and contextual resources to build resilience chronologically. The portraits of this resilience constructing journey will provide a fresh and more informative perspective to our understanding of teacher resilience, and offer implications for teacher education and national policy development. Specifically, this study addresses two research questions.

1. What challenges has the mid-career college EFL teacher been confronted with in the Chinese context?
2. What resilience-building processes has the mid-career college EFL teacher undergone in coping with the challenges?

## Materials and Methods

Narrative inquiry is adopted as the research methodology. Narrative, as an “alternative paradigm for social research” and complementary to experiment, survey, and other methods, appeals more to “likeness” and sense-making by providing a rich description of individuals’ experiences (Barkhuizen et al., 2014, p.1). In the field of education, narrative inquiry has proved especially fruitful in the study of teachers’ professional lives and careers (Clandinin and Connelly, 2000; Barkhuizen et al., 2014).

As a form of qualitative research, it also features being iterative, emergent, and interpretive in terms of data collection, data analysis, and data interpretation (Dörnyei, 2007). It is the systematic exploration by the teacher and for the teacher through her own stories and language (Johnson and Golombe, 2002). Since the researcher is also the participant, this research is self-inquiry and autobiographical. Instead of imposing knowledge on teachers as outsiders, narrative inquiry empowers them to construct knowledge as insiders by looking inward, outward, backward, and forward at their experiences to capture their temporal nature, personal, and social dimensions. Thus, it has potential to create “new sense of meaning and significance” for teachers’ experience within their own professional landscape (Clandinin and Connelly, 2000, p. 4).

### Participant

As a self-narrative inquiry case study, in this research, the participant is the author – LN (the author’s acronym), a mid-career female EF teacher in a Double First-Class University in central China. Based on purposeful sampling, the author meets two selection criteria: (1) she has taught in the frontline for 11years and stepped into a mid-term career phase, with shifting challenges from personal lives and occupational reforms; (2) in spite of adversities, she showed resilience and made above-average achievements in teaching, researching, and managing roles. Her excellence in teaching was measured by evaluation from students and colleagues, and her awards in several high-rank national teaching contests; her achievements in research was judged by research projects she participated in and articles she published; and her managing quality as a deputy director of the College English Department was evaluated by her leaders and colleagues.

To ensure the participant was qualified to be selected as a resilienct teacher, the resilience scale of Connor and Davidson (2003; CD – RISC) was also adopted. The author completed the questionnaire and the result was 82, indicating she was a resilient teacher. Admittedly, the findings from a single-case study cannot be generalized, but they are valuable to reveal “the inner mental worlds of language teacher” and have implications for other teachers and the research at large (Barkhuizen et al., 2014, p. 2).

In the VITAE study, Gu and Day (2013) combined the six professional life phases defined by numbers of years of teaching into three broad groups: early career teachers (0–3 and 4–7years), middle career teachers (8–15 and 16–23years), and late career teachers (24–30 and 31+years). Accordingly, the participant in this study falls into the category of “middle career teachers.” One thing that needs to be noted is that “mid-career teachers,” “middle career teachers,” and “experienced teachers” are often used interchangeably, but these expressions do have the same meaning.

### Data Collection

In order to discover the shifting challenges encountered by the author, and rebuild the bumpy journey of her resilience building, data were collected from the source between her fifth and eleventh teaching years in four sections: (1) Teaching journals were produced by LN after class at the end of each unit, and diaries concerning challenges and resilience-building, some of which were written in her notebook, and others were uploaded online in her blog. (2) Class observations and field notes were written in her notebook or shared in the “moment” of WeChat (an online social networking service, similar to WhatsApp). (3) Chat records with colleagues and students referred to the texts and pictures in WeChat and emails between LN and them, which were mainly in digital format. (4) Research materials included reports and papers in LN’s teaching studies. Overall, the various types of collected data included teaching journals, diaries, class observations, field notes, chat records, and research materials, which helped to enhance the trustworthiness of the study (Gao and Zhang, 2020). Besides these “hard” data, she also relied heavily on the “soft” data – her memory and reflection to gain evidence (Wall, 2008). The data from these sources were triangulated to each other, mapping out a full picture of the teacher’s resilience building. To a large extent, narrative inquiry in this research was her self-inquiry, in which the author came back and forth between two identities as a participant and researcher. As a participant, she acted like an “insider” and fully displayed and shared her highs and lows in her personal, relational, and organizational aspects, providing a richer and rounded understanding of the central character. As a researcher, she stood back like an “outsider” in analyzing and interpreting the data iteratively to construct the contextualized knowledge of the participant. In order to fully convey the “likeness” of the participant’s experience, narrative analysis (storytelling) is mainly adopted in the “findings and discussion” part, and prior studies are referred to make sense of the narratives (Barkhuizen et al., 2014).

### Data Analysis

To improve the rigor and credibility of data analysis, the coding process was done inductively through Nvivo 12, a computer-assisted qualitative data analysis software (CAQDAS; Derakhshan et al., 2021), which provided excellent data management and retrieval facilities that supported data analysis, data interpretation, and writing. To ensure the rigorous and systematic analysis, the data codification and analysis were conducted step by step (Gao and Zhang, 2020; Derakhshan et al., 2021). Firstly, “hard data” like teaching journals, diaries, class observations, field notes, chats records, research reports, and papers were input into Nvivo 12, and were read thoroughly to obtain a general sense of the data. Special attention was given to the challenges confronting LN in her mid-career, and big events and factors influencing her response and decision were highlighted and annotated. Then, the data were read closely and coded openly, with reference to memory (see Appendix 1). During the next step, the codes were constantly compared and grouped into related subthemes. Then with constant comparison and contrast, the recurring themes emerged and were categorized into a “higher-order umbrella term” (Derakhshan et al., 2021, p. 6; see Appendix 2). To refine the themes, rounds of analysis were conducted with repeated reading and constant referring to memories. It is worth noting that this study is autobiographical, so the issue of trustworthiness and “member checking” (Lincoln and Guba, 1985; Derakhshan et al., 2021) between the researcher and the participant bothering other qualitative studies did not arise in this study. As for the data concerning LN’s colleagues and students, codes and themes extracted were sent to them for clarity and accuracy. To minimize investigation bias and enhance the confirmability (Lincoln and Guba, 1985; Derakhshan et al., 2021), an outsider researcher was asked for to audit the whole data analysis process, and disagreement arising in the process was discussed and resolved in negotiation.

## Findings and Discussion

In this section, the major themes extracted from the data regarding the challenges confronting the participant are presented first, namely individual challenges, classroom challenges, institutional challenges, and challenges from persistent national reforms. Then, the themes concerning the resilience-building process are stated on terms of challenges, resources, and response and strategies in the three phases: (1) homeostasis-breaking and changes-resisting, (2) reflecting, recognizing, and rebuilding, and (3) assimilating and adapting. To chart a detailed map of the participant’s uneven resilience-building journey, the nuance and intricacies of her physical, emotional, and cognitive responses and strategies are fleshed out chronologically in her self-narration. Meanwhile, excerpts and commentaries from her story are listed as pieces of evidence of the themes, and theories and prior studies are referred to help the discussion of the findings.

In order to fully convey the “likeness” and verisimilitude of the reality experienced by the participant, the first person “I” will be used, and the genre of narrative writing will be used in this part.

### Challenges Confronting the Experienced Chinese EFL Teacher

#### Individual Challenges

At the age of 30, in my 6th year being an EFL teacher, my orderly life was disrupted by a baby cry. I became a mother. However, the pleasure and curiosity of being a new mother were soon diluted by subsequent challenges.

*In the first half year, every night I was woken up three to six times to feed and care the crying baby, and I was left exhausted and grumpy the next morning (Excerpt from diaries).*

The problem was compounded when my maternity leave ended at the 8th month, which soon made me a juggler caught between being a mother and a teacher. I often felt like being split up, despite the help from my mother and husband, enormous amount of time and energy was required to take care of the baby, so I had to burn the night oil to prepare lessons and grade papers for the next day. What I expected then was this dilemma would be relieved as the baby grew up. However, the reality proved that being a mother indicated an eternal change for female teachers, constant roles shifting and working hard to strike a balance between the two demanding sides.

At different career phases, teachers face different types of stressors and conditions of work and life (Gu and Day, 2007, 2013; Beltman et al., 2011). Unlike novice teachers who may be wholly engaged in teaching, mid-career teachers have a life beyond work, especially a new “center of gravity” or priority for teachers when they have to care for little children or elderly relatives (Jin et al., 2021). Their age and life phase inevitably impact on their wellbeing and approach to work (Jin et al., 2021).

#### Classroom Challenges

*“Is there any volunteer who would like to share your opinions about this topic?” This was the third time I asked or even begged for students to do the oral task in the class. Still, dead silence. Either because of being embarrassed or being bored, they lowered their heads and buried themselves in smartphone flicking (Excerpt from teaching journals).*

Engaging college students in oral tasks has always been tough, and individual factors including personality, motivation, inadequate oral English ability, anxiety, contextual factors like topics, learning tasks, classroom environment, and kinesthetic, auditory, and visual modalities have been identified accounting for students’ reticence and willingness to communicate (WTC; Peng, 2019; Freiermuth and Ito, 2020). Furthermore, positive past experiences with language teachers and foreign peers were also associated with second language learners’ WTC (Freiermuth and Ito, 2020). Meanwhile, students’ mindset is largely shaped by their cultural background as well as their teacher-student interpersonal relationship (Xie and Derakhshan, 2021). Raised in Confucianism culture which promoted modesty, discreetness in words and actions, Chinese students do not value oratory in public speaking as their western counterparts do.

To make things worse, the “screen culture” pervasive in the new millennium generation builds up a new barrier between teachers and learners (Gu and Day, 2013). According to Jin et al. (2021), given the wide access to resources and language models, the role of language teachers as authoritative linguistic models is being challenged increasingly. When doubts and questions arise, they would rather turn to their phones for help than to me, as one of my students said: *“With a phone, knowledge is available to me anytime anywhere” (Excerpt from chat records)*. Equipped with the “knowledge reservoir,” students now are more like a “supervisor” in the class, since my pronunciation, spelling, and content would often be checked online by students, and my mistakes sometimes would be kindly reminded or left unknown to me, which sometimes made me feel like an imposter caught on the spot. As one of my colleagues said, “*We teachers are edged out gradually from the center by information technology*” *(Excerpt from chat records)*. The blended teaching mode mandated by our department since the Covid-19 outbreak meant 20% percent of course tasks were put online and the offline instruction was reduced proportionally. Coupled with this attention loss, the internet directly connects students and learning resources (Bao et al., 2021), various English courses from renowned teachers and universities on MOOC platforms offering numerous options to students and make me worried that I am going to lose my job in the near future.

#### Institutional Challenges

In the first 5years of my teaching career, every week I had at least 20 teaching hours in the class, which was high above the average workload. In the autumn midterm, I even had *4 hours of teaching every day for 40days in a row (Excerpt from teaching journals)*. The heavy workload often made me feel like a “teaching machine,” shuttling from the campus to my apartment day and night, so I had no time to do research for my professional development. Lying exhausted on the bed after work, I often wondered when this machine mode would be over.

In my 8th year, the dream day came. My teaching workload was reduced to 12 hours a week. However, the relief and pleasure brought by the lightened work did not last long. We had a new dean! Unlike the former one who only cared about whether we finished the workload or not, she was very ambitious and demanded all the faculty to aim high in terms of teaching and researching. Inspired by Positive Psychology, she initiated to break the status quo of “being settled” and establish an uplifting and even competing culture in our department. We were even classified as “combustible” or “non-combustible” according to our degree of motivation and commitment. For the young and middle-aged teachers like me, teaching and research targets were set up, and incentives were also taken to push us forward.

Coupled with the demanding dean, our university updated its evaluation and promotion system with higher requirements since it was listed as the Double First-Class University, a project initiated by Chinese authorities for developing a number of world-class universities and disciplines and enhancing Chinese higher education power and international competitiveness. According to the new criteria, in order to be promoted as an associate professor, I need to achieve highly both in terms of teaching and research. For teaching, I had to win at least first prize in the English teaching competitions held provincially or nationally. Although in recent years opportunities to join in the contests are increasing for teachers, it is still very hard for us to win prizes since there are more than 100,000 EFL teachers in China, and many of them are eager to win prizes to accomplish the evaluation requirement. Within our department, there are about 40 young and mid-aged teachers like me who are striving for senior professional titles, and winning prizes looms like a must-do for us, and it is no exception to me. Whenever news about teaching contests is released, dozens of teachers would sign up for it, which seems like a fierce battle without smoke, and this competing atmosphere puts a lot of teachers under great pressure as I learned in private talks with them. The performativity agendas, initiatives, and limited resources may bring stress and anxiety to teachers and even make some teachers marginalized in the institute (Gu and Day, 2013; Zhang et al., 2018).

Besides the requirement for prizes in teaching contests, at least three high-quality papers published in core journals and one provincial research project are required for professional advancement. Ironically, the new promotion regulation still placed our department as a public English teaching institution while we were assessed in the same way as other teachers from research institutions. The unfair treatment experienced by the EFL teachers in professional evaluation and promotion was also found in other studies (Fan et al., 2021).

This is more challenging for me in three aspects. For a start, I have not received systematic and disciplined training on academic research in my college life, so writing papers and applying for research projects were so intimidating that I tossed and turned in bed without knowing where to start. Meanwhile, the heavy workload in my first 5years deprived me of time and energy to read academic journals and do research, which in turn left me with a weak foundation of academic ability and a bleak prospect for professional development. Furthermore, the big imbalance between the limited number of paper publishing outlets and the high demand of publishing made ordinary EFL teachers like me stuck in a dilemma with no way out.

#### Challenges From Persistent National Reforms

*“I tried so hard, but I do not think I can ever catch up with the shifting education trend.” (Excerpt from chat records).*

My colleague Li’s complaint echoed my concern and stress. Actually, whether we teachers welcome it or not, reform and change have been the norm in Chinese foreign language education. The development of Chinese foreign language education has been closely intertwined with Chinese social, economic, and cultural development, and its original aspiration has been and will always be to bring out talents to serve Chinese development and prosperity (Wen and Chang, 2021). Under the influence of globalization, China has gone through revolutionary changes in all aspects in recent decades. In response, rounds of top-down, deep structural reforms have been initiated by the Ministry of Education in EFL area in term of teaching methods, content, and pedagogical principles (Gu and Li, 2013; Fan et al., 2021).

The information technology-integrated teaching and learning mode was around the corner 7years ago, when I had a faint understanding of what it was and how it would influence my teaching career. Even as it came closer to me 3years ago at a conference, when one of my colleagues introduced the Rain Classroom, an online application designed for blended teaching mode, I still frowned and groaned to a colleague next to me, “Do we really need a phone to empower the interaction in the class?” *(Excerpt from chat records)*. My colleague’s instruction soon paid off. More and more young teachers in my department began to try the Rain Classroom out in their classes, while I remained reluctant to dabble in it. Deep down inside, for one thing, I held doubt about the effectiveness of technology to enhance the interaction in the class. For another, I feared my authority and centeredness would be challenged by those fad technology. Actually, my doubt and fear are echoed and researched in the studies. Reforms, especially those managed poorly often disturb the relative stability of teachers’ work, sometimes their beliefs and practices and self-efficacy (Gu and Day, 2007; Gu and Li, 2013). Teaching is increasingly challenging, and teachers are expected to respond to pressures and comply with shifting reforms, which require them to rethink and reconstruct their conventional teaching beliefs about teaching and learning, and build a student-centered and creativity-centered learning culture in their classrooms (Gu and Li, 2013).

Unexpectedly, the outbreak of Covid-19 in 2020 pressed the fast-forward button of online-teaching mode. Whether it was elementary education or higher education, all Chinses students were quarantined at home, and all teachers were required to teach online. Confronted with the challenging circumstances, I had no choice but to try all means to fumble with different online teaching platforms like ZOOM, Dingding, and Tencent to ensure my teaching performance would not suffer in the critical period. As I struggled to cope with online teaching with newly acquired skills, some experts lectured us to collect students’ data and evaluate online teaching and learning, which posed an unprecedented challenge to me since I hardly knew anything about those complicated software as well as the basics of research on assessment and evaluation. As I worried, after the offline class resumed 8months later, the online-assisted blended teaching became a daily routine, and there was no way to steer myself out of it but face the challenge brought by the technology-integrated teaching ways.

When it rains, it pours. As I was at wit’s end trying to put myself together to navigate the blended teaching mode, we were alarmed with an earth-shaking notification that credit hours of our EFL courses would be reduced in 1 or 2years. I can still vividly remember the horror in my colleagues’ eyes at the news, and we were struck dumb altogether because we knew the teaching hours reduction would only marginalize our subject further and thus make it harder to climb on the “professional ladder,” since research funds and projects available for us would be downsized proportionally.

After the disturbance and panic died down a little, we composed ourselves and began to find ways to prepare for the big change. After rounds of consultation with EFL teachers from other universities, it became clear that we had to reduce English for general purpose (EGP) courses and upgrade our curricular system by developing ESP and EAP courses to meet the needs of the country as well as students to cultivate compound talents with specialty and capability (Data from field notes). This pragmatic orientation in foreign language teaching not only brings out rounds of curriculum reforms, but also implies teachers need to update their knowledge and teaching competencies constantly, which will inevitably arouse a sense of insecurity among them (Fan et al., 2021).

The challenge to me was enormous and intimidating. Since I started to work, I had been teaching the College English Listening and Speaking Course for more than a decade, and I had no experience of developing a new course. What new course should I choose? And what procedures should I follow to set it up? Those questions haunted me day and night as I fumbled to find a new direction for my professional development.

Resilience is a dynamic developmental process and presupposes the presence of threat to the status quo, and thus a positive response to conditions of significant adversity (Richardson et al., 1990; Luthar et al., 2000; Gu and Day, 2007; Gu and Li, 2013; Beltman, 2015, 2020). In this sense, changes or challenges from both inside and outside serve as disruptive power that breaks the equilibrium and triggers teachers’ dynamic process of resilience building, with constant interaction between the individual and the environment (Richardson et al., 1990; Gu and Day, 2013). Evidence from empirical work on resilience also reveals that resilience is context specific, a social construction as well as associated with personal attributes (Ungar, 2004). Therefore, teachers’ resilience building will not only be influenced by “the more proximal individual school or classroom context,” but it will also be influenced by “the broader professional work context” (Beltman et al., 2011, p. 190).

### Resilience-Building Process Going Through by the Experienced Chinese EFL Teacher

According to the division of three scenarios in teachers’ life (Gu and Day, 2007), my mid-career professional life phase, specifically the fifth to eleventh working years are zoomed in for analysis and interpretation to lay out an experienced teacher’s bumpy journey of resilience building. This journey is characterized by the fluctuating intensity of concerns and challenges, and complicated interaction between protectors, riskers, and my physical, emotional, and intellectual endeavors. As an extension of prior studies, this research will emphasize how I activated agency to make full use of the protectors to create favorable conditions for myself instead of equating resilience building as exploring protective factors (Kumpfer, 2002). Specifically, based on the resilience model (Richardson et al., 1990) and different degrees of my emotional and intellectual capabilities, the resilience-building journey could roughly be divided into three phases: the first phase of homeostasis breaking and changes resisting, the second phase of reflecting, recognizing, and rebuilding, and the third phase of assimilating and adapting.

#### The First Phase: Homeostasis-Breaking and Changes-Resisting

The watershed moment of my teaching career came with a baby cry. I became a mother at age of 30 in my 6th year. Before it, I was immersed in a “rosy color period” when I had a happy married life and I felt established in teaching after the novice struggling periods. To some extent, I thought I had realized my dream as a teacher that I cruised teaching in my way, and my teaching was highly evaluated by students at the end of the term. The baby’s coming soon proved to be a disrupter. The equilibrium between life and work was broken since caring for a baby was so demanding physically and mentally that I was mostly occupied by babysitting and had little time for lessons preparation during the first 2years. The time for instruction preparation was so much edged out that I had to stay up late to finish my teaching preparation and daily chores (Data from diaries). As a result, the hormone fluctuation of being a mother and juggling between life and work often left me exhausted, memory deteriorated, and grumpy. As I was busy with baby caring, my young colleagues had passed examinations for PhD and embarked on a new journey for further learning. The peer pressure soon threw me in panic, and undetectably I had come to a crossroad: settled for the status quo, or learning further for professional development (Data from diaries). According to the resilience model (Richardson et al., 1990), disruption and adversity are the prerequisite for the dynamic resilience-building process, among which challenges, stressors, and riskers break the organized state of life and stimulate the individual to struggle his way to reorganize his life and resume the balance from the disruptive experience.

Standing at the crossroad, despite my seeming composure, my response and tackling strategies were still far from positive. As Gu and Li (2013) elaborated there were two different retentions for teachers, physical retention which teachers were reduced to due to burnout and attrition, and the quality retention which teachers can sustain passion and commitment despite the challenging circumstances. The teacher’s resilience is an important “quality retention” issue, especially in China where the teaching job is fairly stable and the dropout rate is low (Gu and Li, 2013). My response at the moment was more like the former one, maintaining the status quo without taking initiatives to confront the intimidating challenges. Mostly because my son was still too young, I prioritized his caring over my investment for the future development. Secretly, I hoped I could devote more time and energies to my professional learning to gain groundbreaking achievement as he grew older. Or I was like an ostrich burying my head in the sand, ignoring shifting changes and reforms concerning EFL teaching, holding fast to my old “present, practice, and produce” way of teaching, and hoping it was the best for my students.

Although riskers like role-shifting as a mother and road-taken decision brought unprecedented challenge and stress, thanks to my supportive family and caring students, their help and encouragement acted as protectors which empowered me to maintain my commitment to work and life instead of falling apart like some middle-aged female teachers suffering from postpartum depression. From the moment of the baby’s birth, my husband began to fulfill his role to care for me and the baby, changing diapers, cradling the baby to sleep, and doing the housework. Meanwhile, in order to ensure me to have a multi-nutrient diet, my mother always prepared the tailored meal for me after reading the cookbook carefully (Data from diaries). They had tried all means to make sure the baby and I live healthily and comfortably. Moreover, I was also uplifted emotionally by my students, dozens of whom flooded in my house to visit me and the baby with flowers and loving words. Their physical and emotional support tided me over the most critical and transformational period. As one student wrote in an email,

*“Thank you so much for your encouraging words, Linda. I was really impressed by your great devotion and commitment to us. In order to arouse our attention in spoken English, you even pronounce words with exaggerated facial expressions and intonation. Although my oral English is bad, you still listened to me carefully and commented on it positively. I am so touched that I will study harder and I will not let your down.” (Excerpt from emails).*

This letter made me realize that I can make a difference to them. My commitment to students’ learning and achievement can spark their interest to study even to life. Meanwhile, students’ appreciation and applause to me acted like the best buffer for me from the uncertainty and unpredictability in the teaching career. Their recognition was a great spiritual booster and even a lifesaver whenever I was in stress and burnout, especially when I was doubtful about my ability to keep up with the changing teaching methods. As researchers hold, a teaching job is emotional by nature, and it is not a matter of entirely conveying knowledge and content and using novel teaching methods and techniques (Pishghadam et al., 2019). Good teaching is charged with positive emotions, and good teachers are not merely well-oiled machines, but they are emotional, compassionate beings who enthusiastically interact and connect with their students in an enjoyable learning environment and fill the class with pleasure and creativity (Fredrickson, 2004; Gu and Day, 2007; Xie and Derakhshan, 2021; see Table 1).

**Table 1:** Summary of variables in LN's resilience-building phase 1 (5–7th years).

Resislience downward: Homeastasis-breaking and changes-resisting		
Scenario 1: Personal dimension dominant		
Challenges (riskers)	Resources (proctectors)	Response and strategies
New role as a mother	Vocation commitment	Focusing on family
Lack of work-life balance	Family support	Resisting challenges
Prefessional delimma	Students’ encouragment	Following my old way in teaching

#### The Second Phase: Reflecting, Recognizing, and Rebuilding

My “ostrich” way of escaping the changing reality proved to be a failure. As more and more young colleagues were on their way to a doctorate, the piling peer pressure made me realize I had to confront the “crossroad” question squarely: be settled for the status quo or seek out a way to break through. This is the typical “to be or not to be” question that the mid-career teachers need to answer, and a key watershed of teachers’ long-term perceived effectiveness. Standing at the crossroads of their professional lives, they have to decide whether to pursue career advancement or to remain in the classroom fulfilling the original “call to teach” (Gu and Day, 2007).

Unlike 2years ago, I could not take the baby as an excuse to defend my demotivation since he was already in kindergarten now. It was time to take action. As I decided to prepare for the doctorate examination, a new regulation released by my university destroyed my hope. It stipulated that all in-service teachers like me should not pursue further degree education anymore or we would be laid off. Although I was furious at being deprived of education right, I had no power to resist but accept it. The only way left for me to advance in the career path was to promote my professional title by achieving more in teaching and research. It was no easier, but I decided to confront the challenges positively after serious reflection upon my teaching experience, which consisted of no shining moments except some praise from students. The mediocre performance made me obscure and marginalized like a shadow in my department. I had to work hard to prove myself and I believed I could do it.

God helped those who helped themselves. I would never forget my shining moment on the podium to be awarded the first prize in the FLTRP Star Teacher Contest.

*I was seen! I not only received rounds of applause from more than a thousand EFL experts and teachers on the spot, and my prize winning news was also headlined on my university’s homepage. I was also bombarded with congratulatory messages from friends and colleagues. This great honor boosted my confidence unprecedentedly. (Excerpt from diaries).*

Because this national contest was so challenging, with hundreds of EFL teachers competing for very few prizes that I did not expect I could make it. It proved that my painstaking efforts in reading and writing for the teaching design in the past 3months were paid off, and I had the capacity to achieve in teaching and research as long as I devoted myself wholeheartedly.

Fredrickson (2004) observed that a subset of positive emotions – joy, contentment, interest, and love promoted discovery of novel actions and social bonds, which served to build personal resources and psychological resilience. Strong positive emotions like joy and contentment I experienced from this success largely promoted my self-efficacy, and empowered me to confront difficulties initiatively and adaptively. Meanwhile, my perception of changes and challenges also changed inadvertently, for I began to regard them as opportunities hidden to learn and transform.

In the next half year, when asked by students, I decided to take the challenge without hesitation to mentor them in a provincial contest. As Bandura (2000) observed, when faced with obstacles, those who doubt their capabilities slacken their efforts, while those who have a strong belief in their capabilities redouble their effort to master the challenges. After thousands of hours of discussing, reading and writing, and rehearsing, my two students under my guidance won the first prize in the important English speaking contest – Introducing Henan to the World, another great honor on my CV and booster to my self-efficacy and courage, adding to my intellectual, social, and psychological resources.

As I was immersed in joy and contentment, the new dean talked to me, *“I see you have won prizes in teaching. Why not step off your comfort zone to try a new role, the associate director of the sub-department? What do you think?” (Excerpt from chat records).* I felt greatly flattered but also anxious and pressured. Managing the institute of dozens of teachers was totally new and more challenging to me, since I was introverted and clumsy at communication. Should I accept this offer? Would I be able to manage it well? After days of consideration, I decided to accept this promotion, believing it was an incentive for me to reach out to colleagues to improve our collegiality.

The moment I was assigned the new role, I was tested by its daunting responsibilities, the high-stake ones like clarifying and specifying the teaching content, objectives, and procedures to teachers at the beginning of the term, drafting out examination papers for thousands of students at the end of the term. In order to ensure effectiveness, I sought help and advice from several directors and colleagues. Daily office chores like communicating with colleagues, attending a regular staff meeting, and paper-writing work sometimes made me hard to switch off. Even at home, I was also on “work mode,” working on weekends to finish teaching and non-teaching work. However, I was glad to find my capability at managing and communicating were highly improved with my positive attitude and strenuous efforts. As Gu and Li (2013) found, it is quite common for mid-career teachers to shoulder double responsibilities in the classroom as well as in the office. As they feel more confident and efficacious in teaching, then they enter a stimulating stage where they begin to take on additional responsibilities at work. These responsibilities often bring them greater expectations from themselves and their schools, and more work-life tensions (Gu and Li, 2013).

According to the resilience model (Richardson et al., 1990), after the initial stage of homeostasis breaking from the disruptive factors, individuals will step into the next stage of struggling his way to recover from the adversity and reorganize his life, from which he will be more adept at suitable coping strategies and protective factors. In retrospect, these 2 years were a milestone in my resilience-building journey, with unprecedented changes premising and facilitating my serious reflecting, teaching belief changing, refined coping strategies, and record-broken achievements in my professional development as a teacher, instructor, and a deputy director. Dazzled by the prizes and praise’s halo, I also underwent various trials and pressure physically, intellectually, and psychologically. Preparing for contests and doing the office work both took me a huge amount of time, making me race against the clock before deadlines. The heavy workload and long hours of sitting in front of computers eventually caused me serious backache and sore eyes. Despite my health problem, what I could not bear was my ignorance of EFL teaching and research, so I plunged myself in reading papers and books about pedagogy published in English and Chinese. Additionally, I also suffered from imposter syndrome a little, worrying that if I did not try hard to gain accomplishments equivalent to the previous ones, I would not live up to others’ expectations (Data from diaries). In spite of those riskers, I took the initiatives to confront them positively and harvested honors and competencies in all aspects. These social, emotional, and intellectual resources constructed would further function as reserves that could be drawn on later to improve the odds of successful coping and survival and fuel resilience building (Fredrickson, 2004; see Table 2).

**Table 2:** Summary of variables in LN’s resilience-building phase 2 (8–9th years).

Resislience upward: Reflecting, recognizing, and rebuilding		
Scenario 2: Situated and professional dimension dominant		
Challenges (riskers)	Resources (proctectors)	Response and strategies
Difficulties at managing work	First prize in teaching contest	Perceiving risks as opportunities
Lack of work-life balance	First prize in students guiding	Keeping professional learning
Imposter syndrome	Promotion as the deputy director	Seeking helps from others

#### The Third Phase: Assimilating and Adapting

As I stepped into the 10th year of my teaching career, I felt I was transformed a lot compared with the old me 3years ago when I was so panicked and hesitant at the crossroad. I was equipped with more composure, confidence, and resilience, a reservoir built after I survived the challenges with milestone prizes and honors on the road taken. As resilience was not built in a day, teaching career was inherently filled with shifting changes and trials (Gu and Day, 2013; Day, 2017; Fan et al., 2021; Jin et al., 2021). As I felt relieved and capable of coping with teaching contests and students guiding, greater challenges emerged to the surface:

*How could I make breakthrough in research proposal applying and paper writing? What new extended course should I develop, and how should I develop it? (Excerpt from research materials).*

Meanwhile, our curricular system must be updated since the credit hours of EGP courses were going to be reduced. In retrospect, these challenges dominated the third phase of my resilience-building journey as riskers as well as activators.

Research proposal writing and academic paper writing differed in genre, format, and other aspects, yet they had something in common to me – important but extremely challenging. As a master in translation studies, the lack of strict and systematic education in EFL teaching and researching made me fumble alone in the dark for a long time. The repeated rejection and failures made me so helpless until I joined an academic research team in our institution. To prepare us with the basics of research in EFL teaching and learning, all research members were assigned reading lists and asked to present in the literature discussion regularly. In the heated discussion, I was often inspired by other members’ insights in literature reading and proposal writing (Data from diaries). More importantly, I was so fortunate to join in an important provincial research project about students’ demotivation led by the team leader. I was asked to help collect data by implementing the questionnaire and semi-structured interviews, process data with SPSS, analyze data, and write part of the research project. Although it was far beyond me, I was fueled and motivated greatly by the trustworthiness and close cooperation in the project team that I determined to seek challenges in this process, working around the clock to read literature about motivation studies, watch online courses about SPSS operating, and learn to write the research report (Data from research reports). After 2years of joint efforts by our project team, the research report of this project was highly evaluated and awarded the first prize by the expert committee in Henan Province. Additionally, the academic paper I wrote on students’ coping strategies of demotivation based on this project was also accepted and published successfully.

Overall, what I harvested from this research project was far more than the honor, but growing capabilities in all aspects of academic research from literature reading, data collecting, data processing, and data analyzing, to academic paper writing. Moreover, the supportive help from the research team also provided me a sense of safety, belonging, and warmth on the rough road forward. Teachers can hold more positive views toward research and become research literate by joining research organizations and associations, and receiving research training in their workplace (Derakhshan et al., 2020b). Meanwhile, as Mercer and Gregersen (2020) observed, when in positive states, teachers would be open to thinking more creatively, seeing more options, and connecting with others – all of which help them become better, more effective, and innovative teachers.

In the same vein, the social network and resources enabled me to make a significant breakthrough in course development. Before that, for a long time, I had been haunted by the daunting task to build a new extended course. Despite racking my brain hard, I still had no clue as to the teaching objectives, content, teaching methods, *etc.*, of the new course. One day, several colleagues and I were called into a meeting about the new course.

*“We should set up some new courses to cope with the impending credit hours reduction. I hope you work hard together to make it, and all resources needed will be guaranteed,” the dean said (Excerpt from chat records).*

Inspired and motivated by the dean’s support, we soon established a course-developing team, working together to bring the dream course to a reality. Thanks to the strong support by our institution, we could not only attend online workshops on curriculum development, visit other universities in person to learn about course building and management, but also invite experts to our institution to guide our work (Data from class observations and field notes). Eventually, with the conflation of the dean’s leadership, the institute’s support, our team’s joint efforts, two extended courses were set up and highly evaluated by students, and even landed the great honor of “the First-Class Course” in Henan province. The groundbreaking establishment made me realize that we could make it as long as we work hard as a team, and there was something more I can do to contribute to the curriculum reform. In spite of the full consciousness of the strenuous effort required, I still made a bold move that I would develop another new course integrating the traditional Chinese culture and EFL teaching, in congruence with the Education Bureau’s initiative to implement Chinese culture-based teaching. Unlike before, instead of sitting and waiting for others’ push, I motivated myself to reach out for leaders and colleagues for their support and help. The new course team was soon set up, in 8months, and we worked hard together to design and refine the teaching content and teaching activities after hundreds of hours of reading, discussing, and evaluating. Now, the new course is full-fledged to meet students in the next semester.

As I look back over this period, the challenges to make a breakthrough in academic research and course development were like grains of sand in the oyster, stimulating me to be more responsive and resilient with the ever-changing contexts. At the same time, I was also backed up with various support and resources from the social web interweaved by the dean, our institute, the research team, and the course community. Especially the dean played a significant role to motivate us and support us to move forward when there seemed to be no way out, serving as “weavers of the fabric of resilience initiatives” (Gu and Li, 2013, p. 299). By learning and researching in the communities, teachers establish a sense of belonging and shared responsibilities, develop perceived self-efficacy and resilience, and thrive and flourish socially and professionally (Gu and Day, 2007).

There are precious qualities nurtured by me in the third phase that distinguishes it from the former two phases: agency to seek changes, challenges, and a growing mindset, and I am more adept at protective factors or coping strategies (Richardson et al., 1990). While I was acutely aware that great accomplishments in the research and course establishing would cost me enormous time and energy, unbalanced work and life, and unpredicted difficulties and distress, I also knew these challenges would also serve as stepping stones to stronger abilities and greater accomplishments (Richardson et al., 1990; Fredrickson, 2004; Gu and Day, 2013). So I took the initiatives to assimilate and adapt to the shifting circumstances, and confront these trials whatsoever, and maintained a deep and unwavering commitment physically, emotionally, and intellectually (see Table 3).

Overall, teachers’ capacities to sustain their commitment or resilience are moderated by their professional life phases and mediated by the contexts or “Scenarios” in which they live and work (Gu and Day, 2007). As teachers’ career phases differ, the personal, situated, and professional dimensions of their scenarios are not static but change accordingly. Therefore, teachers often experience fluctuations of different intensity in these three dimensions, which influence their identities and coping abilities (Gu and Day, 2007). In this vein, teachers face distinctively key personal and professional concerns, tensions, and challenges in their different professional life phases. Teachers’ journey to cope with the aforementioned changes and build resilience is arduous and grueling, and their resilience may also fluctuate with the interaction between the changes and challenges from personal and professional aspects, and their intellectual and emotional capacities to deal with these challenges (Gu and Li, 2013).

**Table 3:** Summary of variables in LN’s resilience-building phase 3 (10–11th years).

Resislience upward: Assimilating and adapting		
Scenario 3: Professional dimension dominant		
Challenges (riskers)	Resources (proctectors)	Response and strategies
Credit hours reduction	First prize in the research project	Seeking changes and challenges
Difficulties in academic research	First-Class course award	Building a course-developing team
New role as the course leader	Support from the leader and institute	Establishing growing mindset

## Conclusion

In this research, narrative inquiry was employed to investigate the challenges confronting the mid-career EFL teacher LN, and to map out the detailed and bumpy journey of her resilience building in the three scenarios with distinctive dominant personal, situated, and professional dimensions. Base on the findings, three important observations can be made.

First, the findings confirm the prior studies that the teaching career is physically, emotionally, and intellectually demanding in nature, consisting of severe challenges in certain circumstances as well as uncertain and unpredictable trials in everyday life. Therefore, although these challenges could be disruptors of the equilibrium of teachers’ personal and professional life, they could also turn up as initiators of resilience-building process by activating teachers’ psychological, emotional, and cognitive resources. Additionally, as teachers’ professional career path advances, they will face different concerns, tensions, and influences peculiar to the circumstances, and teachers’ responses and strategies will be influenced by the dimensions dominant in their personal, situated, and professional life.

Second, the research proposes that there is a reciprocal relationship between positive emotions and positive character traits like resilience and provides evidence to the broaden-and-build theory of Fredrickson (2004). The success in big events in LN’s professional life induced positive emotions like joy, contentment, interest, and love, which in turn broadened her response and strategies repertoire and built her enduring psychological, intellectual, and social resources. The broadened repertoire and established resources were significant to the building and improvement of her resilience. With the upward spiral of resilience building, she actively extended her capability boundaries to accept more novel challenges to experience a new round of positive emotions.

Thirdly, in contrast to the findings of the mainstream studies that resilience was an innate psychological attribute, this research finds that its building was the culmination of collective and collaborative endeavors, which involved a complex web of supports and assets from inside and outside, especially the social resources and networks in the critical career phase. Teachers’ resilience is not static that can be accomplished at one stroke; instead, it involves persistent efforts and long-time devotion, and it is dynamic and can fluctuate in levels depending on teachers’ efforts and capacities differ in shifting scenarios.

Accordingly, implications are drawn out for teachers’ resilience building and professional development. For individuals, teachers should have a better understanding of the prospect that the persistent pressure and recurring setbacks on the career path are on a daily basis, and confront them positively to enhance their resilience. For policymakers and teacher educators, attention should be paid to the mid-career teachers whose concerns and pressure had long been neglected, and provide in-service education programs on resilience building to help them well cope with the ever-present challenges. For institutional leaders, support and resources should be provided to mid-career teachers who are at the crossroad of surviving or thriving, to ensure their full commitment to teaching and sustainable professional development.

This study can be further improved by addressing its limitations in future research. First, although the thick description of a single case – such as this one – can be a good starting point for insightful understanding of understudied groups, future studies may as well take a macro-perspective approach such as the large-scare questionnaire to depict a full picture of teacher resilience by focusing on more mid-career teachers in various teaching contexts and circumstances. Second, the trajectory of the experienced EFL teacher resilience-building was mainly a retrospective and interpretative inquiry into the teacher’s past experiences, so future studies can focus on in-service teacher resilience training programs and look into the effect of the intervention to help teachers sustain passion and commitment to teaching.

## Data Availabiltity Statement

The original contributions presented in the study are included in the article/Supplementary Material, further inquiries can be directed to the corresponding author.

## Ethics Statement

The studies involving human participants were reviewed and approved by Academic and Ethical Committee of Henan University. The patients/participants provided their written informed consent to participate in this study. Written informed consent was obtained from the individual(s) for the publication of any potentially identifiable images or data included in this article.

## Author Contributions

LX conceptualized, collected the data, drafted the manuscript, and edited its language before submission to Frontiers in Psychology.

## Funding

This original research was sponsored by the School of Teacher Education of Henan University. The Research Project is entitled “A Study on Chinese EFL Teachers’ Emotion Regulation, Resilience and Their Work Engagement Based on Positive Psychology (Grant No. YB-JFZX-22)”; the research was also sponsored by the Research Funds for Social Sciences in Higher Education of Henan Province, entitled “A Study on the Teaching Methods for Chinese Culture Courses in English to Cultivate Students’ Ability of Telling the Chinese Stories Well” (Grant No. 2022-ZZJH-522), and was supported by the Undergraduate Teaching Research Project administered by Henan University, entitled “Research on the Cultivation of College Students’ Ability of Telling the Chinese Stories Well in the New Era” (Grant No. HDXJJG2020-43).

## Conflict of Interest

The author declares that the research was conducted in the absence of any commercial or financial relationships that could be construed as a potential conflict of interest.

## Publisher’s Note

All claims expressed in this article are solely those of the authors and do not necessarily represent those of their affiliated organizations, or those of the publisher, the editors and the reviewers. Any product that may be evaluated in this article, or claim that may be made by its manufacturer, is not guaranteed or endorsed by the publisher.
