# Supplementary material for: Challenges and Resilience-Building: A Narrative Inquiry Study on a Mid-Career Chinese EFL Teacher
Source: Front Psychol. 2021 Oct 12;12:758925. doi: 10.3389/fpsyg.2021.758925 (PMC8631175; doi:10.3389/fpsyg.2021.758925)
Supplement: Supplementary file 1 [file Data_Sheet_1.docx]

Appendix 1. Examples of coding

**Codes**

**Transcript**

^1^ Today, I had a talk with my course team members. At first, it was only a casual daily conversation. Gradually, we began to talk about our “headache questions” like how to improve students’ interest and engagement in class. One of them showed us several apps she used in her class and encouraged us to have a try. Then the talk naturally lead to our different understanding of language, learning, and even cognitive linguistics. ^2^I was totally engaged in this “movable feast” and greatly inspired by their insights. ^3^I need to reach out more to them, and hope I have more honest and joyful talks with colleagues.

1 community of learning

2 positive emotions

3 response and strategies
