# Supplementary material for: Challenges and Resilience-Building: A Narrative Inquiry Study on a Mid-Career Chinese EFL Teacher
Source: Front Psychol. 2021 Oct 12;12:758925. doi: 10.3389/fpsyg.2021.758925 (PMC8631175; doi:10.3389/fpsyg.2021.758925)
Supplement: Supplementary file 2 [file Data_Sheet_2.docx]

Appendix 2 Examples of codes, sub-themes, and themes

| **Codes** | **Sub-themes** | **Themes** |
| --- | --- | --- |
| Becoming a mother  Feeding and caring the baby  Stay up late  Exhausted  Grumpy and depressed  Preparing lessons  Juggler | Role-shifting  Physical and mental fatigue  Imbalance between life and work | Individual challenges |
| No interest in oral tasks  The matter of face  Flipping phones  Checking words online  Listening English in APPs  Watching MOOC courses  Learning on U-campus | Students’ reticence and boredom  Screen culture  Online learning resources | Classroom challenges |
| Teaching machine  Physical fatigue  Setting high goals  Cultivating competitiveness  Winning prizes in teaching  Publishing papers in core Journals | Heavy workload  Demanding leader  High evaluation criteria | Institutional challenges |
| Cultivating international talents  Moral education  Online teaching  Blended teaching mode  EGP courses minimized  Updating curriculum | Top-down reforms  New teaching modes  Credit hours reduction | National reforms challenges |
